# Digital Health Technology Use by Vietnamese Americans: Cross-Sectional Study

**DOI:** 10.2196/83091

**Published:** 2026-08-14

**Authors:** Richard Qiu, Jinni Tang, Weiming Ke, Nnume Nwankwo, Lauren B Garza, Alexis Le, Angelina P Nguyen

**Affiliations:** 1 P7 Telemetry/Thoracic & Cardiovascular Surgery Department, The University of Texas MD Anderson Cancer Center, 1515 Holcombe Blvd, Houston, TX, 77030, United States, 1 713-792-2121; 2 Healthcare Transformation Initiatives Department, McGovern Medical School at UTHealth Houston, Houston, TX, United States; 3 Baylor University, Louise Herrington School of Nursing, Dallas, TX, United States; 4 Parkland Memorial Hospital, Dallas, TX, United States; 5 Dell Seton Medical Center at the University of Texas, Austin, TX, United States; 6 Dartmouth College, Geisel School of Medicine, Hanover, NH, United States

**Keywords:** digital health technologies, health information technologies, cyber security, trust, social support, cultural competency, Asian Americans, diabetes mellitus

## Abstract

**Background:**

Digital health technologies (DHTs), including mobile health (mHealth) apps and online medical records, are increasingly being used to support self-management and communication with health care professionals among the general population across the United States. However, few studies have explored whether cultural, privacy, and sociodemographic factors may influence DHT adoption among Vietnamese Americans specifically.

**Objective:**

This cross-sectional study aimed to explore DHT use, barriers to adoption, and their associations with social determinants, such as age, sex, social support, and mental health concerns, among Vietnamese Americans.

**Methods:**

Data were collected from a sample of Vietnamese American adults (n=304) through surveys assessing DHT engagement, confidence in medical record security, and social and psychological factors. Descriptive statistics and inferential analyses, including 2-tailed *t* tests and chi-square tests, were used to examine differences across demographic groups.

**Results:**

Many participants had engaged with mHealth tools (253/304, 83.2%), including using mobile devices for health tracking (185/302, 60.9%), and health care professional discussions (194/301, 64.5%). Online medical records were reported to be widely available (230/304, 75.7%), with a large proportion of the participants (182/227, 80.2%) accessing them at least once in the past year. Privacy concerns were a key barrier to using online medical records, with 40.5% (17/42) of nonusers citing security fears, and 26.6% (81/304) reporting withholding health information from health care professionals. The main reasons participants did not access online medical records (45/304, 14.8%) were that they preferred to speak to health care professionals directly (31/44, 70.5%) or did not perceive a need to access their online medical records (30/43, 69.8%). There were no statistically significant differences in sociodemographic characteristics between DHT users and nonusers. There were statistically significant differences in the levels of social support of participants who had varying levels of confidence in the security of their medical records (*F*_2,301_=10.19; *P*<.001; η²=0.063). A greater proportion of male participants reported distrust (ie, being “not confident” in the security of their medical records) than female participants (*χ*^2^_2_=20.8; *P*<.001), indicating a significant association between sex and confidence level.

**Conclusions:**

While DHT adoption is high among Vietnamese Americans, privacy concerns remain a major barrier to full use of online medical records. Health care professionals and public health initiatives should focus on improving trust in medical record security, incorporating culturally tailored education on the benefits of digital health, and leveraging social media and mHealth tools to optimize patient engagement. Developing stronger family and community support may facilitate the adoption of DHTs.

## Introduction

### Background

The rapid advancement of digital health technologies (DHTs) has revolutionized the way health care is delivered and accessed, including mobile health (mHealth) apps, wearable devices, and telemedicine platforms for the general population across the United States [1,2]. These technologies play significant roles in helping manage chronic health conditions, promote preventive care, and improve communication between patients and health care professionals (eg, physicians, nurses, and registered dieticians) among diverse specialties and settings [2]. DHTs, such as mobile phone–based systems and apps, have been shown to be highly effective to lower health care costs [3]. Despite growing DHT use, more than 75% of Asian Americans reported not using these technologies, and they are half as likely as non-Hispanic White individuals to search for health information online [4,5]. Among all Asian Americans, Vietnamese Americans comprise the fourth largest Asian ethnic subgroup nationally [6], yet Vietnamese Americans continue to be an underrepresented population in DHT use and adoption research. Existing studies in this population have been largely qualitative, focused on COVID-19 screening and vaccination; there have been no survey-based studies quantitatively assessing DHT use or its predictors among Vietnamese Americans [7-9]. Vietnamese Americans face unique challenges in health care access, including limited English proficiency, cultural stigma surrounding certain health conditions, and reliance on family-centered decision-making, all of which may affect how they engage with digital health tools [10,11]. There is a paucity of research focusing specifically on Vietnamese Americans, whose cultural beliefs, language preferences, and social dynamics may significantly influence their acceptance of DHTs [10]. Previous studies have also highlighted that trust and privacy concerns are significant barriers to the adoption of DHTs [12,13].

Therefore, the purpose of this study was to explore DHT use among Vietnamese Americans: including mHealth engagement, online medical record adoption, and key barriers to use. This study also assessed potential factors impacting DHT engagement among Vietnamese Americans, such as age, sex, social support, and privacy concerns.

### Theoretical Framework

**Figure 1.**
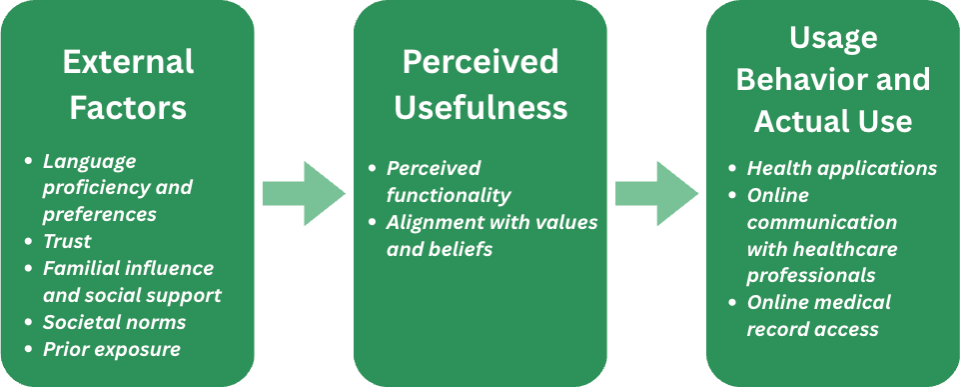
Simplified technology acceptance model for digital health technology use in Vietnamese Americans. Adapted from Venkatesh and Davis [14].

This exploratory study is guided by the extended technology acceptance model (TAM2) to assess the perceived usefulness and actual use of health-related technology by Vietnamese Americans (Figure 1) [14]. TAM2 has been successfully applied to health care technologies, patient portals, mHealth apps, and telehealth, particularly among populations for whom social influence, trust, and accessibility strongly influence technology use [15-17]. In alignment with TAM2, this research explored factors influencing technology adoption, such as language proficiency, trust in health care professionals, family opinions, community norms, and exposure to digital tools through peers or ethnic media. Perceived usefulness indicates facilitators to actual use. Use behaviors, including real-world engagement with DHTs (eg, frequency of online medical records access, health-tracking apps use, and digital patient–health care professional communication), are eventually shaped by both external and contextual factors.

## Methods

### Overview

This paper presents findings from a secondary analysis of a cross-sectional survey study conducted by a coauthor (APN) between November 2023 and March 2024. The methodology for this study has been previously described in detail [18]. Vietnamese Americans are the fourth most populous Asian population in the United States, with the largest concentrations in California, Texas, Washington, Florida, and Georgia [19]. The study included persons who self-identified as Vietnamese American and were aged 18 years or older. Because of the purpose of the original study, interested persons were excluded if they did not have high risk for diabetes (based on a score of 5 or more on the American Diabetes Association Diabetes Risk Test [20]) or a current diagnosis of prediabetes or diabetes.

Initial recruitment efforts were conducted locally at in-person community events in the Dallas-Fort-Worth-Arlington metropolitan area and nationally online through professional nursing organizations and social media groups. Additional participants were referred through snowball sampling. Quota sampling was used to ensure sufficient Vietnamese-language responses for scale validation (ie, at least 10 participants per item on the survey with the highest item count). Bilingual researchers, including the principal investigator and a trained assistant, conducted eligibility screening and data collection in the participant’s choice of either English or Vietnamese language to ensure delivery consistency.

### Ethical Considerations

The study received institutional review board approval from Baylor University (IRBNet ID 2018067‐5) and was conducted in accordance with the Declaration of Helsinki. Informed consent was obtained from all participants prior to data collection, including consent for future analyses. Participant confidentiality was protected through secure data storage and deidentification, with access restricted to authorized researchers and findings reported in aggregate form.

### Measures

The PhenX Toolkit (version 41.5, August 18, 2022) was used as a base for measurement selection in this study [21]. Data collection was conducted in the participant’s choice of English or Vietnamese, and the survey was translated from English to Vietnamese using a cross-cultural adaptation process with initial translations done by a bilingual team member and back-translations done by a certified translation company. Pretesting of translations passed through cognitive interviewing. An expert panel reviewed translations prior to and during validation testing. Participants had the choice of completing the survey face-to-face on paper, by interview, or online by interview or using Qualtrics. Interviews were performed by either the principal investigator or the trained research assistant, both of whom were bilingual.

The primary measure used in this secondary analysis of DHT use over the past 12 months was the Health Information National Trends Survey (HINTS) 2018, cycle 2 [22]. The psychometric properties of the HINTS survey have been evaluated by the National Cancer Institute on a question-by-question basis through expert review and cognitive validity [23,24]. HINTS includes 16 core items, several of which include multiple subitems and built-in skip patterns based on participants’ prior responses (Multimedia Appendix 1). HINTS topics include tablet, smartphone, or electronic device use; internet use (eg, social media, online forums, and YouTube); and online medical records access. Most HINTS items have yes or no response options regarding how participants use DHT. As applicable, the HINTS items also include a third “not applicable” or “don’t know” response option (eg, the item that asks if participants had shared health information from either an electronic monitoring device or smartphone with a health care professional within the 12 months). One HINTS item asks about the confidence level that safeguards, including technological factors, are in place to protect privacy with 3 ordered response options. For participants who had been offered online access to their medical records, items asked who offered this access, how many times the records were accessed, and the usefulness of the records for monitoring health.

Mental health risks were measured with 4 domains of the *DSM-5* (*Diagnostic and Statistical Manual of Mental Disorders* [*Fifth Edition*]) self-rated level 1 cross-cutting symptom measure for anxiety, depression, sleep disturbance, and substance abuse, where a rating of 2 or greater on any 5-point Likert scale item within the domain indicated a risk for that condition. The anxiety, depression, and substance abuse domains, with 2 to 3 items each, have been shown to be internally consistent and valid measures of psychopathology, with a strong sensitivity greater than 0.83 in community settings [25,26]. The sleep domain only consists of 1 item. Collectively, the *DSM* items used in this study demonstrated acceptable reliability (Cronbach α=0.72).

Health literacy was measured by the 18-item Short Assessment of Health Literacy; scores of 14 or less indicate limited health literacy [27]. The Short Assessment of Health Literacy demonstrated acceptable reliability (Cronbach α=0.78). Social support was measured with the total score of 19 five-point Likert scale items from the Medical Outcomes Study (MOS) social support survey, where high scores indicate more support [28]. The MOS social support survey demonstrated good reliability (Cronbach α=0.97). Sociodemographic data such as age, sex, educational level, and household income were also collected.

### Data Analyses

Data were analyzed using SPSS (version 29; IBM Corp). Descriptive statistics were used to describe the sample characteristics and DHT use by Vietnamese Americans. Chi-square tests were used to examine associations between categorical variables where expected counts per cell were at least 5. Independent-samples 2-tailed *t* tests were used to compare mean scores of continuous or ordinal variables when the data were normally distributed, as demonstrated by histograms. Mann-Whitney *U* tests were used when the data for continuous or ordinal variables were not normally distributed.

One-way ANOVAs were also used to assess differences in means across 3 or more groups, for example, comparing social support scores across varying levels of confidence in medical record security. A 2-tailed *P*<.05 was considered statistically significant. Subsequent post hoc test options depended on whether homogeneity of variance was met (Tukey or Sidak post hoc tests) or violated (Games-Howell post hoc test).

## Results

This sample was diverse in age and income, with a mean age of 52.86 (SD 16.37) years and mean household income of nearly US $100,000 (Table 1). The youngest participant was 18 years old and the oldest participant was 85 years old. There were more female participants and highly educated (247/300, 82.3% had completed at least some college education) participants. Most participants reported proficient English language proficiency (228/277, 82.3%) and demonstrated proficient health literacy (256/304, 84.2%). There were no statistically significant differences in sociodemographic characteristics of DHT users versus nonusers.

**Table 1.** Sample characteristics and digital health technology (DHT) use^a^.

Characteristics	Total sample	DHT use	*P* value^b^
Age (y), mean (SD; range)	52.86 (16.37; 18‐85)	52.53 (16.55; 18‐85)	.16
Sex: Male, n (%)^c^	118 (39.1)	108 (38)	.14
Educational level, n (%)^d^			.52
High school or less	53 (17.6)	51 (18.1)	
Some college or bachelor’s degree	184 (61.3)	173 (61.3)	
Graduate degree or higher	63 (21)	58 (20.6)	
Household income (US $)^e^			.93
<50,000	106 (40)	98 (39.4)	
50,000-99,999	57 (21.5)	54 (21.7)	
100,000-149,999	39 (14.7)	38 (15.3)	
≥150,000	63 (23.8)	59 (23.7)	
Health insurance: yes^f^	156 (88.6)	267 (94)	.06
English proficiency: well or very well^g^	228 (82.3)	213 (81.9)	.51
First generation immigrant^h^	247 (81.5)	205 (81.3)	.87
Percentage of life in the United States, mean (% SD; range)	79.7 (19.22; 16-100)	79.8 (19.33; 16-100)	.57
Health literacy level: proficient, n (%)^i^	256 (84.2)	241 (94.1)	.92
Mental health risks ≥2, n (%)^i^	138 (45.4)	130 (45.5)	.93
Social support, mean (SD; range)	73.44 (16.11; 19‐95)	73.78 (15.80; 19‐95)	.14

a Health literacy was measured by the Short Assessment of Health Literacy, where higher scores indicate higher health literacy levels, with scores ≥14 indicating a proficient health literacy level. Social support was measured by the Medical Outcomes Study Social Support Survey, where higher scores indicate more support.

b *P* values represent tests of association between each characteristic and DHT use (user vs nonuser), not comparisons with the total sample.

c Total sample: n=302; DHT use: n=284.

d Total sample: n=300; DHT use: n=282.

e Total sample: n=265; DHT use: n=249.

f Total sample: n=301; DHT use: n=284.

g Total sample: n=277; DHT use: n=260.

h Total sample: n=303; DHT use: n=252.

i Total sample: n=304; DHT use: n=286.

Table 2 presents findings from the HINTS survey. Most participants (289/304, 95.1%) used at least 1 type of DHT in the past year. Many participants (242/304, 79.6%) had used their smartphone or tablet for facilitating discussions with their health care professionals, health tracking, or informing their health decisions. A little more than half of the participants (156/302, 51.7%) had used other health-tracking devices.

**Table 2.** Digital health technology use by Vietnamese Americans.

	Value, n (%)
Any digital health technology tools used within the last 12 months (n=304)	289 (95.1)
Online medical records—general	
Online medical records awareness (n=304)	230 (75.7)
Accessed online medical records at least once in the past year (n=227)	182 (80.2)
Reports online medical records are at least somewhat useful for monitoring health (n=172)	165 (95.9)
Top 3 components of online medical records identified	
Physician visit summary (n=170)	154 (90.6)
Health or medical problem list (n=169)	144 (85.2)
Immunization history (n=171)	145 (84.8)
Top 5 most helpful functions in online medical records	
Fill forms or paperwork related to health care (n=183)	136 (74.3)
Message health care professional (n=182)	122 (67)
Request refill of medications (n=183)	104 (56.8)
Help with deciding how to treat an illness or condition (n=183)	103 (56.3)
Download health information (n=183)	102 (55.7)
Sent online medical information to (n=183)	
Another health care professional	73 (39.9)
Family or caretaker	57 (31.1)
Health service or app	51 (27.9)
Top 2 reasons for not accessing online medical records	
Prefer to speak to health care professional directly (n=44)	31 (70.5)
Did not feel the need to use online records (n=43)	30 (69.8)
Privacy concerns due to online medical records and information withholding	
Nonusers citing security fears (n=42)	17(40.5)
Withheld health information from health care professionals due to privacy concerns (n=304)	81 (26.6)
Health-related uses for smartphones and tablets	
Health care professional discussions (n=301)	194 (64.5)
Health tracking (n=302)	184 (60.9)
Health decisions (n=303)	154 (50.8)
Other health-tracking devices (n=302)	
Used nonphone or nontablet device for health tracking in the past 12 months (eg, Fitbit, glucose meter, or blood pressure monitor)	156 (51.7)

Many participants reported having an online medical record (182/227, 80.2%). Most participants who accessed their online medical records (165/172, 95.9%) reported that online medical records were at least somewhat useful. The top 3 most helpful functions of their online medical records were for filling out forms or paperwork, messaging health care professionals, and requesting medication prescription refills.

The top 3 reasons participants gave for not accessing online medical records were that they preferred to speak to their health care professionals directly, did not feel the need to use online records, and that they reported a lack of trust in the privacy safeguards of online medical records. A total of 40.5% (17/42) of nonusers expressed concern about the security of their health data online. More than one-quarter of all respondents (81/304, 26.6%) even reported withholding information from their health care professionals due to those privacy concerns.

There were statistically significant differences in the levels of social support of participants who had varying levels of confidence in the security of their medical records (*F*_2,301_=10.19; *P*<.001; η^2^=0.063). Participants who were very confident (mean 77.70, SD 13.45) scored significantly higher than those who were somewhat confident (mean 71.94, SD 15.54; *P*=.005) and those who were not confident (mean 65.85, SD 21.80: *P*=.003). The difference between somewhat confident and not confident participants was not statistically significant (*P*=.19). A greater proportion of male participants reported distrust (ie, being “not confident” in the security of their medical records) than female participants (30/118, 25% vs 17/184, 9%; *χ*^2^_2_=20.8; *P*<.001), indicating a significant association between sex and confidence level.

The following nonsignificant trends were found: (1) a smaller proportion of participants with 2 or more mental health risks reported being very confident in the security of their medical records than those with 1 or no mental health risks (45/119, 32.6% vs 74/119, 44.6%; *χ*^2^_2_=5.3; *P*=.07), (2) a larger proportion of participants who reported using tablets or smartphones for health-related reasons also reported receiving disparate care than participants reporting no reported disparate care (53/58, 91% vs 144/173, 83%; *χ*^2^_1_=2.3; *P*=.13), and (3) a larger proportion of participants who did not report proficiency in the English language also reported that they were not confident in the security of online medical records compared with participants who reported proficiency in the English language (11/49, 22% vs 32/228, 14%; *χ*^2^_2_=4.6; *P*=.10).

## Discussion

### Key Findings

The key findings of this study include the high overall DHT engagement of Vietnamese Americans, and a key facilitator was that most participants found DHT to be useful. While DHT adoption was high among Vietnamese Americans in this study, privacy concerns remained a major barrier to full use. There were also interesting demographic and psychosocial differences linked to DHT perceptions and use related to sex and social support.

### Privacy Concerns

Digital divides [29] may deepen if we do not address concerns related to the security of personal health information. For some participants, these concerns ran so deep that they reported withholding information from their health care providers. The proportion of participants who withheld information from their health care professionals in this present study was greater than that reported in other studies [30]. Withholding information can lead to missed diagnoses and delayed treatment, increasing health care inequities for populations such as the Vietnamese Americans [31]. Likewise, the likelihood of withholding personal health information decreases when there is a perceived high quality of care [32]. Although AI offers transformative potential for DHTs by enabling personalized, predictive, and context-aware interventions [33], there is uncertainty regarding how this will further impact the confidence that Vietnamese Americans have in the security of their protected health information online, such as demographic information, physical or mental health conditions, and care provided.

The collectivist cultural values common among Vietnamese Americans [11] must also inform how DHTs are promoted and explained. Tailoring DHT messaging in culturally and linguistically appropriate ways, such as offering Vietnamese-language tutorials, community testimonials, and intergenerational success stories, can improve resonance and reduce perceived risks. These messages should also acknowledge and support collective decision-making norms within families and communities, an often-overlooked factor in digital health adoption in Asian communities [34]. By addressing these specific cultural needs and values, health care professionals can provide more effective and meaningful persuasion in promoting DHT systems.

Recommended strategies to improve privacy, security, and trust in DHTs include compliance with data privacy regulations, robust security measures, and the provision of personalized and digital health care services [35,36]. Building trust in DHT systems among Vietnamese Americans should begin with targeted privacy education that clearly explains how DHTs protect personal health information. Nurses are well-positioned to lead efforts to address patients’ privacy concerns and build trust in DHT systems. For example, a systematic review concluded that DHT was a feasible, effective, and acceptable method for reducing hemoglobin A_1C_ levels in individuals with prediabetes or diabetes, especially when there is timely and responsive personalized coaching by a dedicated health care professional [37].

### Social Support

Participants in this present study who expressed high confidence in the security of their online medical records had significantly stronger social support. This finding suggests that social connectedness may play a role in shaping perceptions of digital privacy and trust, potentially influencing willingness to engage with health technologies. Nurses and public health practitioners can collaborate with trusted community figures and influencers to codevelop campaigns that highlight the safety and usefulness of DHTs in culturally relevant ways. Those with high social support may also feel more confident navigating online systems through help from others [38]. One study suggested that interventions promoting patient engagement via electronic health record portals should incorporate in-person technical support, which may overcome barriers related to privacy and security concerns [2].

While patient-facing strategies are crucial, health care provider behaviors remain a powerful driver of DHT adoption [32]. Health care providers’ endorsement and active promotion of the DHT tools’ benefits can significantly influence patient adoption and sustained use of digital systems. Nurses can serve as digital health ambassadors, reinforcing how technologies enhance patient–health care provider communication, improve care continuity, and facilitate timely access to health data.

### Sociodemographic Factors

Although the present study found no statistically significant differences between DHT users and nonusers, these participants had a relatively high mean household income of nearly US $100,000, and the majority also had proficient health literacy and reported accessing their patient portal at least once in the past year.

Other studies have shown that sociodemographic factors such as household income, English language proficiency, and health literacy may impact DHT use. Nearly half of low-income, limited English proficient Vietnamese Americans from the Lee et al [39] study reported using the internet; but only 28% reported using the internet to log in to access their patient portal. On the other side of the spectrum, Tuitert et al [40] noted that DHT use was higher in participants with a higher income. Studies have also linked limited language proficiency with low health literacy [41]. Limited health literacy has also been associated with difficulty accomplishing tasks on their patient portals (eg, finding their treatment plans) and more basic computer barriers (eg, difficulty using a mouse) [42].

One possible implication of the findings from this study is that higher income, English literacy, and health literacy levels may reduce barriers, such as limited access to devices, unstable internet connectivity, and difficulty navigating online health systems. Greater financial resources may also facilitate ownership of multiple internet-enabled devices and access to paid, higher-speed internet services, while proficient health literacy may enhance users’ ability to locate, understand, and apply health information from digital platforms [42,43]. Taken together, these findings support the notion that digital health adoption is closely linked to broader social determinants of health and that interventions aimed at increasing patient portal use in Vietnamese American communities must address not only technology design but also structural and educational barriers that disproportionately affect lower-income, limited English proficient populations.

### Limitations

While this study drew from a relatively large and diverse Vietnamese American sample, it may be subject to sampling bias due to convenience and snowball sampling. Also, while validated tools and dual-language data collection were used, self-reported data are not as reliable because they may be subject to recall or social desirability bias. For example, the HINTS survey does not ask participants to specify which electronic health record systems or patient portal platforms they had used, which limits the interpretation of findings regarding system-specific features and security perceptions. Furthermore, no formal adjustments for multiple comparisons were applied, and potential confounders were not controlled for, given the exploratory nature of this study,

### Conclusions

This study suggests that for Vietnamese Americans, the digital divide is no longer primarily about access, but about trust and meaningful engagement. Although DHT use is widespread, persistent concerns about privacy and data security limit full use and may even compromise care when patients withhold information. These findings highlight the need to shift digital health efforts toward building trust, improving transparency, and delivering culturally responsive communication. Trust in digital systems is shaped not only by technology but also by social support, cultural values, and health care provider relationships, underscoring the importance of community-informed and health care provider–led interventions.

As digital health continues to expand, especially with emerging technologies such as AI, ensuring that systems are trusted, inclusive, and aligned with patient values will be critical to preventing new forms of inequity. Ultimately, advancing digital health equity will depend not just on who adopts these tools but on how confidently and effectively they are used.

## Supplementary material

10.2196/83091 Multimedia Appendix 1 Operationalized constructs of the extended technology acceptance model from sections B and D of the Health Information National Trends Survey (HINTS).

## Abbreviations

DHT: digital health technology
*DSM-5*: *Diagnostic and Statistical Manual of Mental Disorders, Fifth Edition*
HINTS: Health Information National Trends Survey
MOS: Medical Outcomes Study
TAM2: extended technology acceptance model
