# Supplementary material for: Digital Health Technology Use by Vietnamese Americans: Cross-Sectional Study
Source: Asian Pac Isl Nurs J. 2026 Aug 14;10:e83091. doi: 10.2196/83091 (PMC13475787; doi:10.2196/83091)
Supplement: Supplementary file 1 [file apinj-v10-e83091-s001.docx]

**Appendix A. Operationalized Constructs of the Technology Acceptance Model 2 from Sections B and D of the Health Information National Trends Survey (HINTS) [1]**

| **TAM2 Construct** | **Construct Operationalized** | **HINTS Item Number(s) & Description** | **Variable Type** |
| --- | --- | --- | --- |
| **Actual Use** | Health-related uses for smartphones and tablets | #B8- Yes/no for each option for how participants may have used their tablet or smartphone for health-related reasons | Each option: Categorical (Dichotomous- Y/N) |
|  | Other health tracking devices | #B9- Yes or no item asking if they’ve used an electronic device other than a tablet or smartphone to monitor or track their health | Categorical (Dichotomous- Y/N) |
|  | Any DHT used in the past year | Transformed variable for selecting at least one time for item #D6 and/or any “yes” responses for items #B8a - #B8c, #B9 #B10, #B11b, #B11d, and #B11e, which indicated use of smartphones, tablets, online medical records, or other monitoring devices for health-related reasons | Categorical (Dichotomous- Y/N) |
|  | Accessed online medical records at least once in the past year | #D6- Frequency ranges of accessing online medical record | Interval (Ordinal) transformed to categorical (Dichotomous- Y/N) |
|  | Sent online medical records | #D10- Select all that apply from three listed options for where participants may have sent their online medical information | Each option: Categorical (Dichotomous- Y/N) |
| **Perceived Usefulness** | Online medical records are at least somewhat useful for monitoring health | #D11- Rating for how useful their online medical records are for health monitoring | Interval (Ordinal, Likert-type) transformed to Categorical (Dichotomous- Y/N) |
|  | Helpful Functions Used in Online Medical Records | #D8- Select all that apply from seven listed options for how participants have used their online medical records | Each option: Categorical (Dichotomous- Y/N) |
| **External Factors** | Online medical records awareness | #D8- Yes, no, or don’t know options for item asking if any medical records are maintained in a computerized system | Categorical as described (transformed to Dichotomous- Y/N) |
|  | Confidence level of medical record safeguards | #D2- Three ordered levels of confidence from *very confident* to *somewhat confident* and then *not confident* | Interval (Ordinal) |
|  | Identified Components of Online Medical Records | #D9- Select all that apply from five listed components that are included in their online medical record | Each option: Categorical (Dichotomous- Y/N) |
| **External Factors- Barriers** | Reasons for not accessing online medical records | #D7- Select all that apply for five listed options for reasons why participants have not accessed their online medical records and an additional “other” option | Each option: Categorical (Dichotomous- Y/N) |
|  | Non-user’s privacy concerns due to online medical records | #D7d- This was a listed option for reason why participants have not accessed their online medical records | Categorical (Dichotomous- Y/N) |

1.	National Institutes of Health USDoHaHS. Health Information National Trends Survey Annotated Form, Cycle 2, Full-content, English version. 2018.
